# A Novel Method to Represent the Three-Dimensional Inclination of the Distal Radius Joint Surface

**DOI:** 10.3390/diagnostics15030345

**Published:** 2025-02-01

**Authors:** Akira Ikumi, Reo Asai, Yusuke Eda, Tooru Uchida, Sho Kohyama, Takeshi Ogawa, Yuichi Yoshii

**Affiliations:** 1 Department of Orthopedic Surgery, Institute of Medicine, University of Tsukuba, Tsukuba 305-8577, Ibaraki, Japan; 2 Department of Orthopedic Surgery, Tsukuba Medical Center Hospital, Tsukuba 305-8558, Ibaraki, Japan; 3 Department of Orthopedic Surgery, Tokyo Medical University Ibaraki Medical Center, Ami 300-0395, Ibaraki, Japan; 4 Department of Orthopedic Surgery, Kikkoman General Hospital, Noda 278-0005, Chiba, Japan; 5 Department of Orthopedic Surgery, National Hospital Organization Mito Medical Center, Ibaraki 311-3193, Ibaraki, Japan

**Keywords:** distal radius, three-dimensional analysis, joint inclination, rotational alignment, computed tomography

## Abstract

**Objectives:** This study aims to define three-dimensional (3D) parameters for the inclination of the distal radius joint surface. The goal is to develop standardized parameters for fracture reduction through comprehensive 3D evaluations of the joint surfaces. **Methods:** We analyzed 112 CT scans of unaffected wrists (56 males and 56 females) to construct 3D models of the distal radius. Using 3D coordinates, the normal vectors and angles were calculated based on three reference points on the distal radius joint surface. These normal vector components were then converted into unit vector components A, B, and C for the x, y, and z axes, respectively. Additionally, the angles of these unit vectors were assessed in the xy, yz, and xz planes. The 3D measurements were compared between males and females and against traditional two-dimensional (2D) parameters such as palmar tilt and radial inclination. **Results:** For males, the unit vector components were as follows: A: −0.14 ± 0.09, B: −0.92 ± 0.02, and C: −0.36 ± 0.07; for females, A: −0.21 ± 0.08, B: −0.90 ± 0.03, and C: −0.36 ± 0.05. Significant differences were found between males and females for the A and B vector components (representing the palmar–dorsal and proximal–distal axes, *p* < 0.01). The angles of the unit vectors in the xy, yz, and xz planes were 8.9 ± 5.4°/12.9 ± 5.0°, 21.3 ± 4.1°/22.1 ± 3.2°, and 22.2 ± 14.8°/28.8 ± 10.1° for males and females, respectively. There were significant differences between males and females in the angles of the xy and xz planes (sagittal and axial planes, *p* < 0.01). Strong correlations were observed between the xy-plane vectors and palmar tilt (r = 0.96), as well as between the yz-plane vectors and radial inclination (r = 0.88). **Conclusions:** This study evaluated the 3D inclination of the distal radius joint surface, revealing significant gender differences. This method, which also allows for the assessment of rotational alignment—difficult with conventional techniques—is expected to be a key 3D parameter in treating distal radius fractures.

## 1. Introduction

Three-dimensional (3D) assessment of the distal radius joint surface using computed tomography (CT) has emerged as a valuable tool in clinical practice, providing detailed insights into bone morphology and fracture characteristics [1,2,3]. Compared to traditional two-dimensional (2D) imaging techniques, 3D evaluations offer superior accuracy, particularly for understanding complex joint geometries and rotational alignment. Despite significant advancements, there remains a critical gap in establishing standardized 3D parameters that define the standard morphology of the distal radius joint surface. Addressing this gap is essential for improving fracture reduction precision and developing gender-specific treatment approaches.

Recent studies have highlighted gender-based differences in the shape and dimensions of the distal radius joint surface, with males typically having larger and more robust bony structures compared to females [4,5,6,7]. These anatomical differences can affect fracture patterns, surgical techniques, and outcomes, particularly in terms of plate positioning and screw insertion angles [8,9,10], highlighting the need for gender-specific reference values in clinical assessment.

Previous research has introduced foundational methods for 3D assessment of the distal radius, including identifying key reference points, defining baseline coordinates, and establishing 3D parameters such as palmar tilt and radial inclination [11,12,13]. This foundational work is essential for accurately assessing the directions of fracture dislocations and the conditions of fracture reduction. In our previous study [11], 3D parameters for palmar tilt (3D-PT) and radial inclination (3D-RI) were defined, which are comparable to the radiographic 2D parameters of palmar tilt and radial inclination. However, these studies have predominantly focused on converting 3D measurements into 2D parameters. There is limited work addressing the joint’s inclination and rotational alignment in a comprehensive 3D context, which is critical for evaluating the true orientation of the joint surface. Defining 3D parameters for the standard morphology of the distal radius based on gender-specific variations is expected to enhance the evaluation and precision of fracture reduction and related procedures. Therefore, we tried to establish a method to express joint angles in 3D by analyzing the vector of the joint reference plane.

The objective of this study is to define novel 3D parameters for the inclination of the distal radius joint surface and to establish standardized values that account for gender-specific anatomical variations. We hypothesized that significant gender-based differences exist in the inclination of the distal radius joint surface and that these differences can be captured and quantified using advanced 3D imaging techniques to enhance the assessment of joint rotation.

This article is structured as follows: Section 2 outlines the imaging protocols, participant selection criteria, and analytical approaches used to develop and validate the 3D parameters. Section 3 presents quantitative findings, highlighting gender-specific differences and correlations with traditional 2D parameters. Section 4 evaluates the clinical implications of these findings, emphasizing the advantages of 3D measurement techniques over conventional methods. Finally, Section 5 summarizes the key contributions and potential future applications of this research.

## 2. Methods

The study adhered to the principles outlined in the Declaration of Helsinki and received approval from the institutional review board (approval number T2022-0041). This study was conducted in accordance with the STROBE (Strengthening the Reporting of Observational Studies in Epidemiology) guidelines for reporting observational studies. This retrospective case–control study (Level III evidence) involved reviewing a radiographic database to identify CT scans of normal wrists. Regarding the informed consent process, the data used in this retrospective study were reviewed under an “opt-out” consent policy, in accordance with our institutional ethics guidelines. This policy allowed for the use of patient data collected during routine clinical care without the need to re-contact participants for additional consent, thereby avoiding the burdensome process of re-consent for each participant included in the study. From the database, we selected CT images of unaffected wrists taken for comparison with injured wrists. The absence of previous symptoms or injuries in the unaffected wrists was verified through patient interviews and medical records. A total of 112 wrist CT scans were analyzed, with 56 male and 56 female participants, age-matched (males: 19–95 years, average age 56.0 years; females: 18–93 years, average age 60.1 years). Age matching was performed using the individual matching method. Male–female combinations with an age difference of 5 years or less were selected based on the participants’ CT data list. Exclusion criteria included patients with a history of traumatic arm injuries and individuals younger than 18 years.

### 2.1. Three-Dimensional Bone Morphology and Analysis

CT imaging and 3D model analysis of the distal radius were conducted using previously established protocols. CT images were obtained with settings of 120 kV and 100 mAs [14], a section thickness of 1–1.5 mm, and a pixel size of 0.3 × 0.3 mm (Sensation Cardiac, Siemens, Munich, Germany). The CT scans were performed with the forearm in a neutral position, covering from the metacarpal bone level to approximately 13 cm proximal to the radius joint surface. The 3D bone model of the distal radius was analyzed using BoneSimulator software (Orthree, Osaka, Japan, https://www.e-radfan.com/product/7255/). Image data were imported into the software, where 3D surface models of the distal radius were generated using a surface construction algorithm [14,15].

A coordinate system was established based on the 3D data of the distal radius. The long axis of the radius was calculated by identifying the proximal-to-distal center curve of the radial shaft from various cross sections. The central point at each level of the radial diaphysis surface was calculated, and the approximate straight line through these points was defined as the long axis of the radius. This long axis was designated as the *y*-axis, with the proximal direction as positive and the distal direction as negative. The *z*-axis was determined by the orthogonal projection of a line from the base of the sigmoid notch to the radial styloid process, with the radial direction considered positive and the ulnar direction negative. The *x*-axis was perpendicular to the yz-plane, with the palmar direction positive and the dorsal direction negative. The yz, xy, and xz planes were defined as the coronal, sagittal, and axial planes, respectively. The coordinate origin was set at the intersection of the joint surface and the long axis of the radius.

**Figure 1:**
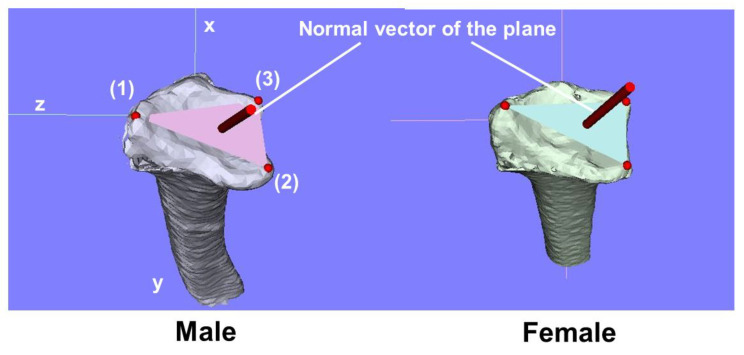
Representative images for each reference point and normal vector. (1) Radial styloid process, (2) Volar edge of the sigmoid notch, (3) Dorsal edge of the sigmoid notch.

Three reference points were marked on the 3D model: (1) the radial styloid process, (2) the volar edge of the sigmoid notch, and (3) the dorsal edge of the sigmoid notch, according to the previous definition. The 3D coordinates of each point and the barycenter of the plane formed by these three points were evaluated. The area of the plane enclosed by the three reference points was also measured. Additionally, the normal vector of this plane was calculated using the equation Ax + By + Cz + D = 0, and its components (A, B, and C) were converted to unit vector components (Figure 1).

**Figure 2:**
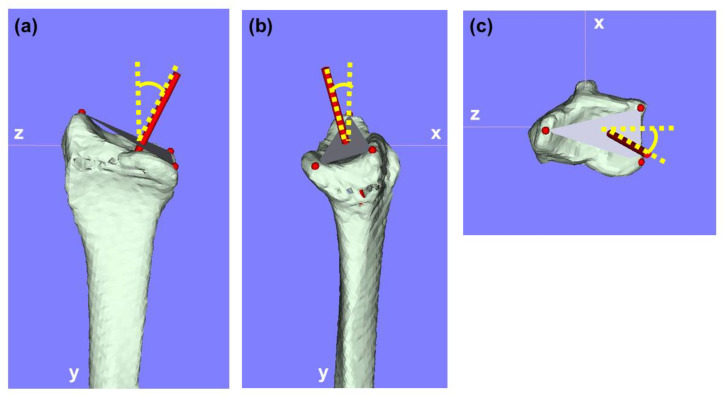
Angles of vectors in the xy, yz, and xz planes. (**a**) Angle of vector in the yz-plane. (**b**) Angle of vector in the xy-plane. (**c**) Angle of vector in the xz-plane.

To compare 3D parameters with conventional X-ray measurements, the angle between the line from reference point (2) to reference point (3) and a line perpendicular to the long axis of the radius was measured as the 3D-PT in the sagittal view. Similarly, the 3D-RI was measured as the angle between the line from reference point (1) to reference point (2) and a line perpendicular to the radius’s long axis in the coronal view. Angles of vectors in the yz-plane, xy-plane, and xz-plane were also measured and compared between 3D-PT and 3D-RI (Figure 2).

### 2.2. Statistical Analysis

The results are presented as mean ± standard deviation. To test the normality of datasets, the Shapiro–Wilk test was used. Differences between males and females were compared using a Welch’s *t*-test with respect to the distances from the origin for the three reference points, the barycenter, the plane area connecting the three reference points, the unit vector components, and the vector angles of each plane. Correlation between the vector angles of the xy, yz, and xz planes were evaluated using Pearson’s correlation coefficient. Correlations between angles of vectors in the yz and xy planes and 3D-PT and 3R-RI were also evaluated using Pearson’s correlation coefficient. All analyses were performed using IBM SPSS Statistics version 29 (IBM, Tokyo, Japan), and *p*-values of less than 0.05 were considered significant.

## 3. Results

**Figure 3:**
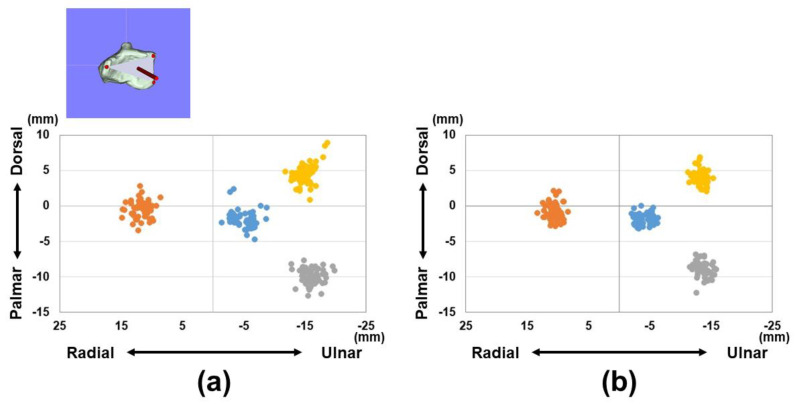
Coordinates of three reference points in the axial plane (yz-plane). (**a**) Results of coordinates for males. (**b**) Results of coordinates for females. Orange dots indicate the radial styloid process: reference point (1). Gray dots indicate the sigmoid notch volar edge: reference point (2). Yellow dots indicate the sigmoid notch dorsal edge: reference point (3). Blue dots indicate the barycenter.

**Figure 4:**
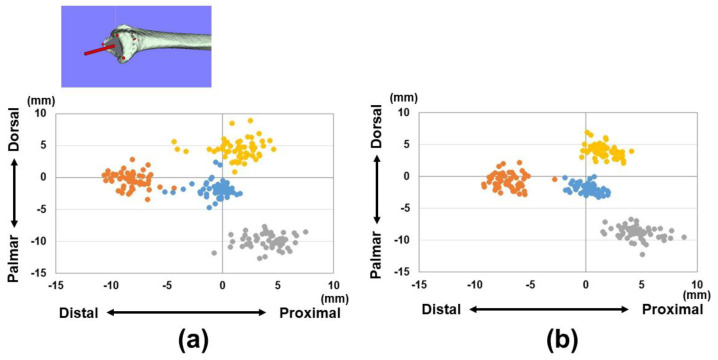
Coordinates of three reference points in the sagittal plane (xy-plane). (**a**) Results of coordinates for males. (**b**) Results of coordinates for females. Orange dots indicate the radial styloid process: reference point (1). Gray dots indicate the sigmoid notch volar edge: reference point (2). Yellow dots indicate the sigmoid notch dorsal edge: reference point (3). Blue dots indicate the barycenter.

**Figure 5:**
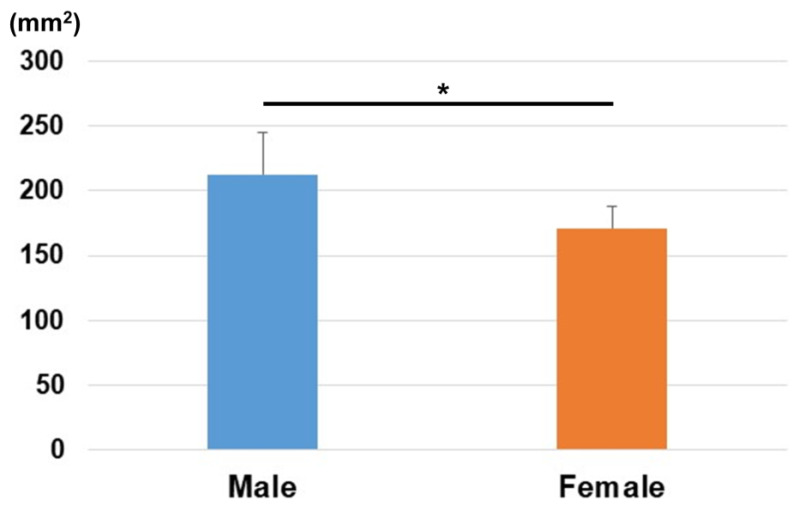
The area of the plane enclosed by the three reference points. The area was significantly larger in males than in females (*: *p* < 0.01).

The distances from the origin for the reference points (1)–(3) and barycenter are shown in Table 1 and Figure 3 and Figure 4. Variations in each reference point were larger in the sagittal direction than in the axial direction. The positions of each reference point from the origin were located at (1) 14.3 ± 1.4/12.6 ± 1.0 mm for the distal–palmar–radial position, (2) 19.4 ± 1.4/16.9 ± 1.2 mm for the proximal–palmar–ulnar position, (3) 15.7 ± 1.6/14.1 ± 0.8 mm for the proximal–dorsal–ulnar position, and (barycenter) 5.6 ± 1.7/4.7 ± 1.1 mm for the proximal–volar–ulnar position for males and females, respectively. Significant differences were observed in all reference points between males and females (*p* < 0.01). The plane areas were 212.6 ± 32.6 mm^2^ and 170.8 ± 17.4 mm^2^ for males and females, respectively. There were significantly larger areas in the male group compared to the female group (*p* < 0.01) (Figure 5).

**Figure 6:**
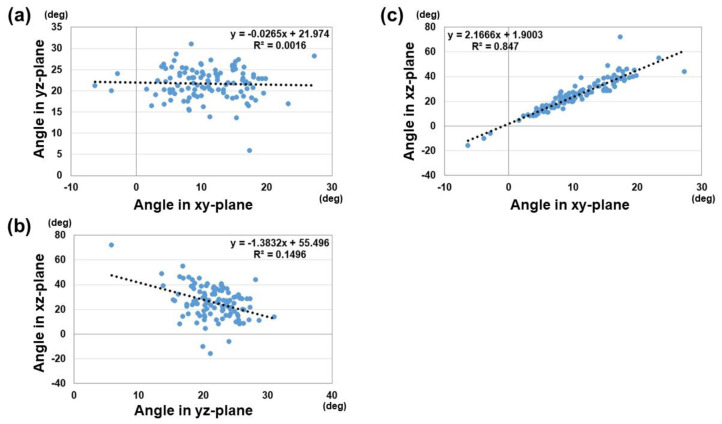
Correlation between the vector angles of the xy, yz, and xz planes. (**a**) Correlation between the vector angles of the xy-plane and yz-plane. (**b**) Correlation between the vector angles of the yz-plane and xz-plane. (**c**) Correlation between the vector angles of the xy-plane and xz-plane.

**Figure 7:**
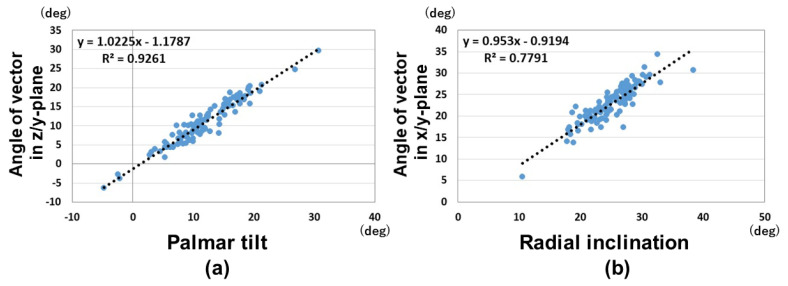
Correlations between the vector angles of the xy and yz planes and 3D-PT and 3D-RI. (**a**) Correlation between the vector angle of the xy-plane and 3D-PT. (**b**) Correlation between the vector angle of yz-plane and 3D-RI.

The results of unit vector components and the angle parameters are shown in Table 2. For the unit vector components, there were significant differences between males and females for the components of A and B (*p* < 0.01). This suggests that the normal vectors of distal radius joint surface were different in the palmar–dorsal direction and proximal–distal direction for males and females. For the angle parameters, there were significant differences between males and females in the angles of the xy and xz planes (*p* < 0.01). The variation of the angle in the xz-plane is larger than of the angles in other planes. A weak correlation was observed between the vector angles of the xz-plane and yz-plane (correlation coefficient: 0.39). A strong correlation was observed between the vector angles of the xy-plane and yz-plane (correlation coefficient: 0.92) (Figure 6). The correlation coefficient between the yz-vector and 3D-PT was 0.96, and between the xy-vector and 3D-RI 0.88 (Figure 7).

## 4. Discussion

3D measurement revealed a clear difference in the inclination of the articular surface of the distal radius between males and females in this study. This 3D analysis method helps to evaluate the standard angles for the distal radius joint surface. The vectors in the xy and yz planes were well correlated with palmar tilt and radial inclination. In addition, we could use a parameter to evaluate the alignment in the xz-plane, which was previously difficult to achieve using 2D parameters. This method is useful for providing parameters of rotational alignment in the axial plane as well as conventional palmar tilt and radial inclination in the sagittal and coronal planes.

The use of 3D measurements in distal radius joint surface analysis is highly advantageous compared to traditional 2D or X-ray-based methods. X-ray imaging, while useful for general bone structure, often lacks accuracy in assessing the complex geometry of bone fractures, particularly for rotational alignment and fine angular adjustments. In contrast, 3D measurements offer a detailed and spatially accurate view, allowing clinicians to assess bone morphology and fracture planes in their true orientation, which is rarely possible with 2D projections due to limitations in the image angles [16]. 3D imaging significantly reduces measurement errors by capturing the complete geometry of the bone and avoiding distortions caused by projection effects seen in X-rays [17,18]. This leads to more precise preoperative planning and improves outcomes in surgical interventions, particularly when fixing fractures. In fact, studies have demonstrated that the standard deviation of measurements using 3D models is considerably lower than that from X-ray-based measurements, leading to more consistent and reliable data across different observers and settings [19]. Moreover, 3D-assisted surgical planning, including patient-specific implants, allows for a higher degree of personalization in treatment, improving the alignment and fixation of fractures, which is crucial for the recovery of joint function [20]. Thus, the application of 3D measurements in clinical settings provides a more robust tool for addressing distal radius fractures, enhancing both the accuracy of the procedure and the recovery process.

When examining the relationship between vector angles, a strong correlation was found between the angles in the xy-plane and yz-plane. This suggests that individuals with a greater palmar tilt have joint surfaces in external rotation. A previous study indicated that as the volar tilt decreases, load transmission shifts from volar to dorsal and from the lunate fossa to the scaphoid fossa [21]. Conversely, with an increase in palmar tilt, the radial styloid process moves relatively dorsally, potentially increasing load transmission through the lunate fossa. It is also known that even under the same stress, fracture patterns can vary [22]. These anatomical shape differences may influence fracture patterns. Various concepts have been proposed so far regarding fracture patterns of distal radius fractures using CT images, and fixation techniques have been suggested based on these fracture patterns [23,24]. By comparing fracture patterns with unaffected models, it may be possible to predict the type of fracture that is more likely to occur in an individual.

One of the strengths of this study is the high correlation observed between the vectors in the xy and yz planes with the 3D-RI and 3D-PT. These correlations validate the accuracy and reliability of this 3D analysis method for assessing joint inclination compared to the conventional method. Furthermore, the additional parameter provided by this method, the evaluation of alignment in the xz-plane, introduces a new dimension of rotational alignment assessment in the axial plane. Rotational alignment in the axial plane has been difficult to evaluate in traditional 2D evaluations using radiographs [25,26,27,28]. This capability complements the conventional parameters of palmar tilt and radial inclination in the sagittal and coronal planes, offering a more comprehensive evaluation of the distal radius alignment.

This study has several limitations. Firstly, the retrospective nature of the study and the exclusive use of CT images from unaffected wrists may limit the generalizability of the findings to actual fracture cases. Although symptom- and trauma-free unaffected wrists were extracted during the data selection process, the large age variation cannot rule out the possibility of selection bias in the data. Secondly, the 3D image analysis and registration process, while innovative, is complex and requires advanced imaging technology and software, which may restrict its widespread clinical use, especially in resource-limited settings. It may be necessary to develop applications for easy measurement. Finally, further research is needed to evaluate how the use of these values in surgical practice affects clinical outcomes, while this study establishes standard values.

## 5. Conclusions

The 3D assessment method developed in this study provides a novel and comprehensive approach to evaluating the inclination and rotational alignment of the distal radius joint surface. The standard values established for both males and females offer valuable reference points that can enhance the accuracy of fracture reduction and fixation procedures. Future research should focus on validating these findings in clinical practice and further exploring the clinical implications of gender-based anatomical variations in distal radius morphology.

## Figures and Tables

**Table 1:** **3D coordinates of the reference points and barycenter and distance from the origin.** For the values shown in bold, there were significant differences between males and females with *p*-values less than 0.01.

	Male				Female			
Avg. (SD) (mm)	x	y	z	Total	X	y	z	Total
Reference points								
1	−0.5 (1.2)	−**8.2 (1.3)**	**11.6 (1.4)**	**14.3 (1.4)**	−0.8 (1.1)	−**7.0 (1.2)**	**10.5** **(1.0)**	**12.6** **(1.0)**
2	−**10.0 (1.1)**	4.1 (1.6)	−**16.2 (1.5)**	**19.4 (1.4)**	−**8.9 (1.0)**	4.6 (1.5)	−**13.7** **(1.0)**	**16.9** **(1.2)**
3	4.4 (1.5)	1.4 (1.8)	−**15.0 (1.4)**	**15.7 (1.6)**	4.0 (1.0)	1.6 (1.1)	−**13.4** **(0.9)**	**14.1** **(0.8)**
Barycenter	−2.0 (1.2)	−**0.5 (1.3)**	−**5.0 (1.7)**	**5.6 (1.7)**	−1.9 (0.7)	**0.2 (0.9)**	−**4.1** **(1.2)**	**4.7** **(1.1)**

**Table 2:** **Results of unit vector components and angle parameters.** Asterisks show the significant differences of the parameters between males and females. °; degree, *; *p* < 0.05.

**Parameters**	**Male**	**Female**
Vector components		
A	−0.14 ± 0.09	−0.21 ± 0.08 *
B	−0.92 ± 0.02	−0.90 ± 0.03 *
C	−0.36 ± 0.07	−0.36 ± 0.05
Angles of vectors		
xy-plane	8.9 ± 5.4°	12.9 ± 5.0° *
yz-plane	21.3 ± 4.1°	22.1 ± 3.2°
xz-plane	22.2 ± 14.8°	28.8 ± 10.1° *
Angle parameters		
3D-RI	24.0 ± 3.9°	25.9 ± 3.7°
3D-PT	10.6 ± 5.4°	13.4 ± 5.3° *

## Data Availability

The datasets analyzed during the present study are available from the corresponding author upon reasonable request.
